# Relation of *IL28B* Gene Polymorphism with Biochemical and Histological Features in Hepatitis C Virus-Induced Liver Disease

**DOI:** 10.1371/journal.pone.0037998

**Published:** 2012-05-29

**Authors:** José A. Agúndez, Elena García-Martin, María L. Maestro, Francisca Cuenca, Carmen Martínez, Luis Ortega, Miguel Carballo, Marta Vidaurreta, Marta Agreda, Gabriela Díaz-Zelaya, Avelina Suárez, Manuel Díaz-Rubio, José M. Ladero

**Affiliations:** 1 Department of Pharmacology, University of Extremadura, Cáceres, Spain; 2 Department of Biochemistry & Molecular Biology, University of Extremadura, Cáceres, Spain; 3 Genomics Unit, Service of Clinical Biochemistry, Hospital Clínico San Carlos, Instituto de Investigación Sanitaria del Hospital Clínico San Carlos (IdISSC), Madrid, Spain; 4 Liver Unit, Service of Gastroenterology, Hospital Clínico San Carlos, Instituto de Investigación Sanitaria del Hospital Clínico San Carlos (IdISSC), Madrid, Spain; 5 Service of Pathology, Hospital Clínico San Carlos, Instituto de Investigación Sanitaria del Hospital Clínico San Carlos (IdISSC), Madrid, Spain; 6 Laboratory of Molecular Genetics, Hospital de Terrassa, Terrassa (Barcelona), Spain; 7 Service of Clinical Microbiology, Hospital Clínico San Carlos (IdISCC), Madrid, Spain; 8 Liver Unit, Service of Gastroenterology, Hospital Clínico San Carlos, Department of Medicine, Medical School, Universidad Complutense, Instituto de Investigación Sanitaria del Hospital Clínico San Carlos (IdISSC), Madrid, Spain; Rosalind Franklin University of Medicine and Science, United States of America

## Abstract

**Background/Aims:**

Polymorphism at the *IL28B* gene may modify the course of hepatitis C virus (HCV) chronic infection. Our aim was to study the influence of *IL28B* rs12979860 gene polymorphism on the biochemistry and pathology of HCV-induced disease in the clinical course from mild chronic hepatitis C to hepatocellular carcinoma.

**Methods:**

We have determined the rs12979860 single nucleotide polymorphism (SNP) upstream *IL28B* gene in two groups of patients with HCV-induced chronic liver disease: 1) 268 patients (159 men) with biopsy-proven chronic hepatitis C, to analyse its relation with biochemical, virological and histological features; and 2) 134 patients (97 men) with HCV-related hepatocellular carcinoma. The distribution of the analysed SNP in hepatocellular carcinoma patients was compared with that found in untreated chronic hepatitis C patients. All patients were white and most were Spaniards.

**Results:**

In multivariate analysis ALT values were higher (*P* = 0.001) and GGT values were lower (*P*<0.001) in chronic hepatitis C patients homozygotes for the major rs12979860C allele as compared with carriers of the mutated rs12979860T allele. Steatosis was more frequent (Odds ratio = 1.764, 95% C.I. 1.053–2.955) and severe (*P* = 0.026) in carriers of the rs12979860T allele. No relation was found between the analysed SNP and METAVIR scores for necroinflammation and fibrosis, and there were no differences in the distribution of the analysed SNP between hepatocellular carcinoma and untreated chronic hepatitis C patients.

**Conclusion:**

The *IL28B* rs12979860 polymorphism correlates with the biochemical activity and the presence and severity of liver steatosis in chronic hepatitis C.

## Introduction

The natural history of the infection with hepatitis C virus (HCV) is variable and difficult to define. Firstly, the proportion of infected patients that clear the virus in the acute phase of the infection broadly oscillates between 14% and 46% of cases [1]. Long-term follow up studies in patients with chronic hepatitis C have shown a great variability in the rate of progression of the liver disease, although after 2 or more decades many infected persons develop progressive hepatic fibrosis and cirrhosis, that is related to a high risk of suffering from hepatocellular carcinoma (HCC). There are some well-known host-related factors that are clearly associated with a more rapid and severe progression of the liver disease, as when it is acquired at an older age [2], and the metabolic syndrome complex (non-alcoholic liver steatosis, obesity and type II diabetes mellitus) [3]. Data on a more benign course in female gender are controversial [4], [5]. There are some reports on host genetic factors related to the risk of acute HCV infection becoming chronic [6] and to the rate of progression in the chronic phase, as are some human leukocyte antigen class II antigens [7], [8] and polymorphisms in the genes of transforming growth factor-β_1_ [9] and angiotensin II [10].

A genome wide association study (GWAS) has identified a non-coding single nucleotide polymorphism (SNP), rs12979860, that resides 3 kb upstream of the *IL28B* gene, located in the long arm of chromosome 19, which encodes IFN-λ3 [11]. Two other GWAS identified a second non-coding SNP, rs8099917, located 7.5 kb upstream of the *IL28B* start codón [12], [13]. Both SNPs are in partial linkage disequilibrium and are part of an *IL28B* haplotype that shows ethnic variations and is a strong determinant of response (or lack of response) to therapy for HCV chronic infection [14], [15].

The mechanisms that regulate the influence of *IL28B* polymorphisms on the success rate of therapy in chronic hepatitis C remain unknown. These genetic traits are not related with levels of intrahepatic *IL28B* gene expression but the baseline expression of interferon stimulated genes (ISGs) is significantly higher in patients carrying the minor rs8099917G allele [16], [17], that is in linkage disequilibrium with the minor rs12979860T allele, thus letting a narrower margin of response when HCV acutely infects the liver or when interferon-based therapy is instituted in chronically infected patients [18]. These findings may explain why the spontaneous clearance of HCV virus is more frequent in subjects with the rs12979860CC genotype [19]–[21] and that this genotype and the linked rs8099917TT genotype are predictors of SVR, as unanimously confirm several studies [11]–[13], [16], [22]–[28].

In spite of the high number of studies mentioned above, data on the influence of the *IL28B* gene polymorphism on the natural history of untreated chronic HCV infection are scarce. Abe *et al.* [29] found that Japanese patients carrying the rs8099917TT genotype had lower plasma levels of gamma glutamyl transferase (GGT) and showed more severe necro-inflammation and fibrosis scores in liver biopsies as compared to carriers of the minor G allele, but the weak (0.01<p<0.05) association of the histological scores with the *IL28B* polymorphism needs independent confirmation.

*IL28B* polymorphism-related differences in the baseline immune response to HCV infection may influence the severity of liver necro-inflammation, the main stimulus for fibrogenesis that is the hallmark of an unfavourable course of chronic hepatitis C.

The aim of this study was to investigate the distribution of the rs12979860 SNP upstream of the *IL28B* gene in a well-phenotyped population of chronically HCV-infected patients covering the whole spectrum of disease severity, from null-mild fibrosis to hepatocellular carcinoma, to elucidate whether this polymorphic trait may be related with the course of chronic HCV infection.

## Materials and Methods

Starting by October 1997 we collected and stored peripheral blood for DNA extraction from all HCV-infected patients referred to our Liver Unit who gave informed consent according with the Declaration of Helsinki. The study was approved by the local ethics committee of the Hospital Clínico San Carlos, Madrid, Spain. The study design included two series of patients: 1) patients with a diagnosis of chronic hepatitis C and positive HCV viremia who were scheduled for their first liver biopsy and naïve for antiviral therapy; and 2) patients with a diagnosis of hepatocellular carcinoma (HCC) on HCV-induced liver cirrhosis in accordance with current criteria [30]. All HCC patients were considered as cirrhotic because most of them were diagnosed with liver cirrhosis before the diagnosis of CHC and the clinical study of the remaining ones was consistent with cirrhosis. Moreover, hepatocellular carcinoma is exceptional in HCV-induced non-cirrhotic chronic liver disease [1]. A blood sample was taken for standard laboratory tests and DNA extraction on the same day of the liver biopsy in patients of the group 1, and when a definite HCC diagnosis was established in patients of the group 2. Patients with HIV or active HBV infections were excluded. A database was created to include clinical and demographical data, and baseline biochemical, virological and histological results.

### Ethics Statement

All patients provided informed consent according with the Declaration of Helsinki. The study was approved by the local ethics committee of the Hospital Clínico San Carlos, Madrid, Spain. This consent was verbal, after providing to patients with full explanation of the objectives and methods of the study, guarantying them absolute privacity in accordance with the Spanish legal rules, that are more strict than those in other European countries. This circumstance was communicated to the Ethics Commmittee before obtaining its authorization. In addition, the determination of the IL28B polymorphism is a routine method at the clinical laboratory of our centre since april2011.

### Laboratory Methods

Quantitative analysis of HCV-RNA was performed with the Cobas Amplicor HCV Monitor version 2.0 (Roche Molecular Diagnostic). The detection range was 600 IU/mL to 8.5×10^5^ IU/mL. Starting from July 2005, viral RNA was extracted automatically using Cobas AmpliPrep, and the viral load was detected using Real-Time polymerase chain reaction (PCR) using Cobas TaqMan (Roche Diagnostics) which has a detection range of between 10 IU/mL and 2×10^8^ IU/mL [31]. Viral load was classified as low (<400000 IU/ml) or high (≥400000 IU/ml), according with Witthöft *et al*. [32] HCV genotypes were determined by a reverse hybridization assay (INNO-LiPA; Innogenetics). The genotypes are assigned on the basis of sequence variations in the 5′untranslated region of HCV following gene amplification using reverse transcription polymerase chain reaction (RT-PCR).

Liver biopsy specimens obtained from patients of group 1 were examined by the same pathologist. Necroinflammation grade and fibrosis stage were scored using the METAVIR system [33]. To perform some analysis, necroinflammation was classified as null-significant (0–2) versus severe (3). Fibrosis was coded as non-mild fibrosis (F0–F1) versus moderate-advanced fibrosis (F2–F4). Steatosis of the liver was evaluated according with the Brunt score [34]: absent or grade 0 (<5% of hepatocytes affected), grade 1 (5–33% of hepatocytes affected), grade 2 (33–66% of hepatocytes affected); and grade 3 (>66% of hepatocytes affected).

*IL28B genotyping* was carried out by means of custom TaqMan Assay (Applied Biosciences Hispania, Alcobendas, Madrid, Spain) designed to detect the rs12979860 SNP. The detection was carried out by qPCR in an Eppendorf realplex thermocycler by using fluorescent probes. The amplification conditions were as follows: After a denaturation time of 10 min at 96°C, 45 cycles of 92°C 15 sec 60°C 90 sec were carried out and fluorescence was measured at the end of every cycle and at endpoint. All samples were determined by triplicate and genotypes were assigned both, by the gene identification software (RealPlex 2.0, Eppendorf) and by analysis of the reference cycle number for each fluorescence curve, calculated by the use of CalQPlex algorithm (Eppendorf). For technical validation purposes, the amplified fragments for twenty individuals carrying the rs12979860 C/C and C/T genotypes and eleven individuals with the T/T genotype were sequenced, and in all cases the genotypes fully corresponded with those detected with fluorescent probes.

### Statistical Analysis

Continuous variables, expressed as mean (SD), were compared with the Student’ *t* test or the Mann-Whitney *U* test, each when adequate, depending on their Gaussian distribution. A p value <0.05 was considered significant. Categorical variables were compared with the χ^2^ or the Fisher exact tests, each when appropriate, and the effect of differences was established by calculating the odds ratio with the 95% confidence interval.

The variables different at a p value <0.05 in the univariate analysis were included in a multivariate analysis based on a logistic regression model to identify which ones were independently related to the determined SNP in the vicinity of the *IL28B* gene.

## Results

### Group 1 (Chronic Hepatitis C)

Two hundred and sixty eight patients were included. Patient profiles are shown in Table 1. Most patients were men (59.3%). All patients were Caucasian, mostly Spaniards. One hundred and seven (39.9%) carried the rs12979860CC homozygous genotype, whereas the remaining 161 were homo- (33 patients, 12.3%) or heterozygotes (128 patients, 47.8%) for the T allele. The studied polymorphism was in Hardy-Weinberg equilibrium.

**Table 1:** Analysis of biochemical and virological parameters in relation with the rs12979860 genotype in 268 patients with chronic hepatitis C.

Parameter	All patients(268 cases)	CC genotype(107 cases)	T allele carriers(161 cases)	*P* (univariate)	*P* (multivariate)
Age (yrs.)	47.2 (10.5)	45.9 (10.4)	48.3 (10.5)	0.076	
Gender (M/F)	159/109	66/41	93/68	OR = 1.17795% C.I. = 0.714–.940	
Viral load ≤/>400.000 IU/mL/n.a.	48/213/7	20/83/4	28/130/3	OR = 1.11895% C.I. = 0.592–2.114	
Viral genotype 1/non 1/n.a.	225/40/3	84/20/3	141/20/0	OR = 0.59695% C.I. = 0.303–1.171	
Hb (g/dL)	15.1 (1.4)	15.0 (1.4)	15.2 (1.4)	0.399	
Platelets (10^9^/L)	202 (59)	202 (57)	203 (61)	0.830	
Bilirubin (mg/dL)	0.83 (0.45)	0.78 (0.36)	0.87 (0.50)	0.135	
AST (IU)	67 (48)	71 (47)	64 (48)	0.224	
ALT (IU)	112 (94)	130 (103)	99 (85)	**0.012**	**0.001**
AST/ALT	0.69 (0.28)	0.64 (0.24)	0.73 (0.30)	**0.002**	0.593
GGT (IU)	79 (118)	47 (42)	100 (146)	**<0.001**	**<0.001**
Alkaline phosphatase (IU)	111 (59)	111 (54)	111 (62)	0.986	
Cholesterol (mg/dL)	178 (36)	184 (40)	173 (31)	**0.022**	0.102

Major allele: C. Minor allele: T.

n.a.: not available.

Biochemical baseline data that showed differences at a p value <0.05 when comparing carriers of the rs12979860CC genotype with carriers of the rs12979860CT/TT genotypes were ALT (0.012), AST/ALT ratio (p = 0.002), GGT (p<0.001), and serum cholesterol (p = 0.022). In the multivariate analysis only lower values for GGT (p<0.001) and higher values for ALT (p = 0.001) were related with the 12979860CC genotype. (Table 1)

Necroinflammatory grade and fibrosis stage were not related with the rs12979860 genotype. (Table 2).

**Table 2:** Analysis of histological features in relation with the rs12979860 genotype in 268 patients with chronic hepatitis C.

Feature	CC genotype(107 cases)	T allele carriers(161 cases)	StatisticsCC *vs.* T carriers	CT genotype(128 cases)	TT genotype(33 cases)	StatisticsCT *vs.* TT
METAVIR necroinflammatory grade(0/1/2/3)	1/16/40/50	3/30/68/60	Chi ^2^ ^LT^ = 2.409(*P* = 0.121)	2/21/57/48	1/9/11/12	Chi ^2^ ^LT^ = 0.952(*P* = 0.329)
METAVIR fibrosis stage (0/1/2/3/4)	21/33/17/28/8	28/56/27/35/15	Chi ^2^ ^LT^ = 0.0002 (*P* = 0.989)	20/46/22/27/13	8/10/5/8/2	Chi ^2^ ^LT^ = 0.466 (*P = *0.495)
Steatosis (Brunt score 0/1/2/3/n.a.)	74/27/6/0/0	89/56/10/4/2	Chi ^2^ ^LT^ = 4.946 (*P* = 0.026)	71/43/9/4/1	14/13/1/0/1	Chi ^2^ ^LT^ = 0.066(*P = *0.798)
Steatosis (Brunt score) 0/>0/n.a.	74/33/0	89/70/2	O.R. = 1.76495% C.I. = 1.053–2.955	71/56/1	14/14/1	O.R. = 1.26895% C.I. = 0.559–2.877

Major allele: C. Minor allele: T.

n.a. = not available.

Chi^2LT^: Chi square for linear trend.

Liver steatosis was present in a greater percentage of carriers of the T allele than in CC homozygotes (44.0% *vs.* 30.8%, Odds ratio = 1.764, 95% C.I. = 1.053–2.955) and there was a significant trend towards higher grades of steatosis in carriers of the T allele (Chi^2^ for trend = 4.946, *P* = 0.026). (Table 2). When the analysis was restricted to patients infected by HCV genotype 1, similar results were obtained (45.1% *vs.* 32.9%, Odds ratio = 1.618, 95% C.I. = 0.925–2.834, and Chi^2^ for trend = 3.934, *P* = 0.047).

METAVIR necroinflammatory grade was significantly correlated at a *P*<0.001 for Spearman’s *rho* test with serum ALT (alanine amino transferase) and GGT values, but not with the remaining biochemical variables included in the analysis. METAVIR fibrosis stage correlated at the same level of significance with the inverse of platelet count and with serum AST (aspartate amino transferase), ALT, GGT and ferritin values.

### Group 2 (HCV-related Hepatocellular Carcinoma)

One hundred and thirty four patients (97 males, mean age at diagnosis 67.4 years, SD 9.4, range 41–88) were included in this group. All were white Spaniards. The diagnosis of HCC was based on the pathological exam of surgical biopsy or cytological aspirate in 73 patients, and according with current imaging criteria [30] in the remaining 61. All patients in this group had detectable titers of anti-HCV antibodies in serum. Only two patients had been previously treated with interferon-ribavirin before developing HCC.

Fifty two patients (38.8%) carried the rs12979860CC homozygous genotype, whereas the remaining 82 were homo- (16 patients, 11.9%) or heterozygotes (66 patients, 49.3%) for the T allele. (Table 3).

**Table 3:** Distribution of the *IL28B* gene rs12979860 genotype in untreated patients with chronic hepatitis C (CHC) and in patients with HCV-related hepatocellular carcinoma (HCC).

*IL28B* genotype	Group		Statistics
	CHC98 patients (%)	HCC134 patients (%)	
CC	37 (37.8)	52 (38.8)	
CT	49 (50.0)	66 (49.3)	χ^2^ = 0.041
TT	11 (11.2)	16 (11.9)	*P* = 0.979
T allele frequency	0.386	0.366	O.R. = 0.98595% C.I. = 0.672–1.445

Former ethanol use ≥ 40 g/day was reported by 52 patients (50 men). The distribution of the analyzed SNP was very similar as comparing these patients with moderate drinkers or teetotallers (*P*>0.80 for each comparison, data not shown).

### Comparative Analysis of HCV-infected Patients with and without HCC

To evaluate the possible relation between the analyzed *IL28B* SNP and the stage of the HCV-induced liver disease, the CHC patients in the chronic hepatitis group who had not received antiviral therapy after performing the liver biopsy were compared with the group of patients with hepatocellular carcinoma. No significant differences were found between both groups in the distribution of the studied *IL28B* gene SNP (Table 3).

## Discussion

The relation between genetic polymorphisms in the vicinity of the *IL28* gene and the response to pegylated interferon and ribavirin combined therapy for hepatitis C has been clearly demonstrated since the rs12979860 SNP was identified. Several GWAS studies independently disclosed that the possession of any of the homozygous genotypes rs12979860CC or rs8099917TT, which are in strong linkage disequilibrium, greatly increases the probability to obtain sustained viral response after antiviral treatment. [11]–[13], [16], [22]–[28].

However, information on the possible influence of these polymorphisms on the course of hepatitis C infection is presently very limited and incomplete. It has been shown that the carriers of the favourable genotypes have a higher rate of spontaneous clearance of HCV during the acute phase of the infection [19]–[21]. In this study we have shown that GGT levels are lower in patients with chronic hepatitis C that carry the rs12979860CC genotype than in homo- or heterozygotes for the T mutated allele. Abe *et al.* [29], in a cohort of 364 adult Japanese patients with chronic HCV infection, reported a similar and highly significant (*p = *0.001) association between the rs8099917TT genotype and GGT lower values, a link that could be explained because both traits are predictive of a favourable response to therapy.

In addition, we have found that rs12979860CC genotype is associated with higher serum ALT than the remaining genotypes, a finding that is in agreement with data of Thompson *et al*. [35] obtained from a GWAS study that disclosed that only the rs12979860 SNP was significantly associated with baseline ALT levels. Serum ALT is considered as a marker of necroinflammatory activity in the liver as confirms the analysis of our data that shows a linear increase of ALT levels related to METAVIR activity score. These findings are contradictory with those reported by Abe *et al.* [29] who found that ALT and AST levels were lower in carrier of the rs8099917TT genotype, although these authors do not provide the level of significance for this association.

However, our results do not confirm the suggested relation between the *IL28B* gene polymorphism and the histological necroinflammatory activity reported by Abe *et al.* [29] and Thompson *et al.* [35]. Most patients included in the present study were infected with HCV genotype 1. Hence, we have not been able to confirm the interesting results reported by Bochud et al. [36] linking the rs8099917G allele, that is associated with poor response to therapy, with lower necroinflammatory activity (p = 0.04) and milder fibrosis (p = 0.02) in patients infected with non-1 HCV genotypes.

The relation between *IL28B* polymorphism and the stage of fibrosis is controversial. Abe *et al.* [29] found higher fibrosis METAVIR scores among carriers of the rs8099917TT genotype. However, Di Marco et al. [37] in a group of 131 patients with thalassemia major and chronic HCV infection who underwent a liver biopsy reported that older age and the carrier state of the minor alleles at rs12979860 and rs8099917 sites were associated with more severe liver fibrosis (*p*<0.001 and *p<*0.005, respectively). Falleti et al. [38] in a group of 629 HCV-positive patients, disclosed that subjects with the rs12979860TT homozygous genotype had a mean fibrosis score higher than the remaining CC or CT genotypes (*p*< 0.05). Nevertheless, Thompson *et al*. [39] reported that the rs12979860 polymorphism was not associated with advanced hepatic fibrosis in CHC, a finding that is in agreement with our results that are far from the statistical significance for the determined SNP.

Fabris *et al.* [40] analysed the distribution of rs12979860 C and T alleles in a miscellaneous group of Italian patients with chronic liver diseases, most HCV-related, including patients with mild hepatitis (Ishak staging score ≤ 2), cirrhosis and HCC. They concluded that the T allele is more prevalent in HCV-induced cirrhosis than in cirrhosis from other causes (p<0.005) but not when comparing HCV-induced cirrhosis with milder hepatitis C (p = 0.09).

Hepatic steatosis is a frequent finding in chronic hepatitis C, as a probable consequence of a disturbance of lipid metabolism [41]. Our study shows that steatosis is significantly more frequent in CHC patients carrying the mutated T allele, in agreement with two previous studies. Tillmann et al [42] analysed two independent cohorts of patients with a global size of 325 patients with chronic hepatitis C; steatosis was present in 28 of 88 rs12979860CC genotype patients (31.8%) and in 139 of 237 patients carrying the rs12979860T allele (58.6%). These differences were highly significant when each group was analysed separately (as did the authors) or when considering them as a unique group (Odds ratio = 3.04, 95% C.I. = 1.81–5.10). In the second study [43] that included 153 Japanese patients, the analysis of the rs8099917 genotype yielded similar results. A new aspect disclosed in our study is that steatosis is not only more frequent, but also more severe in patients carrying the mutated T allele.

Hepatocellular carcinoma (CHC) represents the most advanced step in the natural course of chronic hepatitis C infection that fortunately is reached only by a minority of patients. If the hypothesis of the existence of any relationship between the *IL28B* genetic polymorphism and the severity of HCV-induced liver disease holds true, the most logical finding should be the existence of differences in the genotype frequencies among the different stages of the disease. We have found no differences in the frequencies of the *IL28B* genotypes as comparing a group of HCV-induced HCC patients with a group of 98 untreated patients with CHC. Our results are coincidental with those reported by other groups, both in Caucasian [36] and in Japanese [44], [45] populations. However, Asahina *et al.* [46] reported that carriers of the rs8099917 TT genotype had a higher rate of sustained viral response to interferon-based therapy (as expected) and that this genotype was associated with a lower incidence of HCC, both in sustained viral responders and in non-responders. A direct effect of the *IL28B* polymorphism on the risk of HCC may not be inferred from these results, as SVR clearly improves the natural course of chronic HCV infection [47], [48] and, among non-responders, the effect of the *IL28B* polymorphism was indirect and associated with slightly lower levels of ALT and alpha-feto protein. Eurich et al. [49], in a study of patients who received a liver graft due to severe HCV-induced liver disease, found that the 12979860TT genotype was more frequent in the 61 patients with HCC on the explanted liver than in the 106 patients without HCC (p = 0.041), suggesting that the major C allele plays a protective role against the development of HCC. In addition, Ren *et al.* [50] found that the rs12979860 T allele was related to the susceptibility of both chronic hepatitis B virus (HBV) infection and HBV-related HCC in a group of Chinese patients. Thus, controversy remains about the possible relation between the *IL28B* gene polymorphism and the risk of developing HCC.

We conclude that the *IL28B* rs12979860CC genotype in patients with chronic hepatitis C is related with higher serum ALT, that is considered as a marker of hepatic necroinflammation, and with lower values of GGT, which increase is a surrogate marker of liver fibrosis and a negative predictive factor of response to therapy [51]. In addition, the carrier state of the rs12979860 T allele is associated with greater frequency and severity of hepatic steatosis in chronic hepatitis C. However, we have not found any relation among this polymorphism and necroinflammatory grade and fibrosis stage directly shown by the liver biopsy, neither with the risk of developing HCV-related hepatocellular carcinoma.
